# Insulin at 100 years – is rebalancing its action key to fighting obesity-related disease?

**DOI:** 10.1242/dmm.049340

**Published:** 2021-11-29

**Authors:** Gemma V. Brierley, Robert K. Semple

**Affiliations:** 1 Biomedical Research Group, School of Life Sciences, Anglia Ruskin University, Cambridge CB1 1PT, UK; 2 The University of Cambridge Metabolic Research Laboratories, Wellcome-MRC Institute of Metabolic Science, Addenbrooke's Hospital, Cambridge CB2 0QQ, UK; 3 Centre for Cardiovascular Science, University of Edinburgh, Edinburgh EH16 4TJ, UK

## Abstract

One hundred years ago, insulin was purified and administered to people with diabetes to lower blood glucose, suppress ketogenesis and save lives. A century later, insulin resistance (IR) lies at the heart of the obesity-related disease pandemic. Multiple observations attest that IR syndrome is an amalgamation of gain and loss of insulin action, suggesting that IR is a misnomer. This misapprehension is reinforced by shortcomings in common model systems and is particularly pronounced for the tissue growth disorders associated with IR. It is necessary to move away from conceptualisation of IR as a pure state of impaired insulin action and to appreciate that, in the long term, insulin can harm as well as cure. The mixed state of gain and loss of insulin action, and its relationship to perturbed insulin-like growth factor (IGF) action, should be interrogated more fully in models recapitulating human disease. Only then may the potential of rebalancing insulin action, rather than simply increasing global insulin signalling, finally be appreciated.

## Abstract (precis)

**Summary:** Insulin resistance drives obesity-related diseases, but it is a complex state featuring loss and gain of insulin action, and dysregulated crosstalk with growth factor signalling. ‘Rebalancing’ signalling may offer a beneficial addition to current therapy.

## Introduction

A century ago, insulin was purified from canine pancreata and administered to people with diabetes mellitus. Until then, diabetes was often a rapidly fatal, wasting illness, commonly of childhood onset. Insulin therapy immediately transformed clinical outcomes. It led to emaciated children rapidly regaining fat and muscle, their longevity becoming measured in decades rather than months (Bliss, 1982). The ensuing years have built on this triumph, as insulin was first sequenced (Sanger, 1959) and its three-dimensional structure determined (Hodgkin, 1972), before recombinant human insulin was synthesised and rolled out in clinical practice. Insulin's primary structure has now been rationally manipulated to yield a portfolio of insulin analogues with kinetics optimised for exogenous use. This has been allied with transformative developments in real-time glucose sensing, with invention of devices able to modulate minute-by-minute insulin delivery, and with deployment of sophisticated algorithms to link such glucose sensing to insulin delivery.

In the face of these extraordinary advances, it is tempting to view the century of insulin as one of unalloyed good news. Yet here the picture becomes more complicated. Within 10 years of the first use of insulin, it emerged that it did not have the same glucose-lowering effect in all patients (Himsworth, 1936; Root, 1929), and the notion of insulin insensitivity or insulin resistance (IR) was born. Decades later, development of radioimmunoassay confirmed that insulin concentrations are high, not low, in many people with type 2 diabetes (Yalow and Berson, 1960a,b). The tissue receptor for insulin, known only biochemically for many years (Kasuga et al., 1982; Freychet et al., 1971), was fully revealed in 1985 when the sequence of the insulin receptor (*INSR*) gene was reported (Ullrich et al., 1985; Ebina et al., 1985). This confirmed the receptor to be a transmembrane tyrosine kinase. Three years after this discovery, people with extreme IR who had loss-of-function mutations in *INSR* were described (Kadowaki et al., 1988; Yoshimasa et al., 1988).

The core anatomy of postreceptor signalling was elucidated over the next two decades (White and Kahn, 2021; Haeusler et al., 2018). This was, for some time, commonly characterised as a bifurcating cascade leading to mitogenic signalling through the RAS/RAF/MEK/ERK pathway and predominantly metabolic signalling through phosphoinositide 3-kinase (PI3K). This simple view is no longer sustainable, however. For example, somatic mutations activating the PI3K signalling pathway are among the most common driver mutations in cancer and other growth disorders (Madsen et al., 2018), and co-operation between RAS and PI3K signalling is well documented (Castellano and Downward, 2011). Indeed, further signalling subpathways have been identified downstream of INSR, and a complex array of intra- and interpathway crosstalk and feedback has been identified (White and Kahn, 2021; Accili et al., 1996). Further challenging notions of the linear insulin signalling pathway, some evidence suggests that receptor tyrosine kinases, far from being static cell surface receptors that solely initiate signalling cascades, can themselves form transcriptional complexes within the nucleus (Goldfine and Smith, 1976; Hancock et al., 2019). Intracellular insulin signalling is now best viewed as being mediated by a dynamic network of signalling and trafficking events (Accili et al., 1996).

Concerted use over many years of global and conditional murine knockout models has addressed the nature and consequences of IR (Biddinger and Kahn, 2006). Although these studies have provided a valuable framework for understanding the core principles of insulin action *in vivo*, there are major differences in murine and human development, metabolism and propensity to develop IR-related tissue pathology. This problem is compounded by the ‘glucocentric’ focus of much historical IR literature (McGarry, 1992), and also by organisation of clinical care into different specialities for people with different IR-related conditions. This means that many important mechanistic aspects of human IR and its relationships to organ pathology in humans remain to be understood.

## IR or insulin action imbalance?

IR is usually defined by reduced ability of insulin to lower blood glucose. Diabetes only occurs when there is failure to maintain insulin secretion in the face of the increased demand. Many people, usually with obesity, are in a state of compensated IR, in which IR is balanced by increased blood insulin concentration, or hyperinsulinaemia. IR in type 2 diabetes is only partially compensated, with insulin concentration high, but not high enough to normalise blood glucose. In humans, examining the consequences of complete insulin deficiency (untreated type 1 diabetes), or compensated IR without diabetes, may be particularly helpful in trying to determine which IR-associated conditions are caused by IR with high insulin concentration and which are merely associated with it, or caused by lack of insulin action.

Absolute deficiency of insulin action causes severe hyperglycaemia due to unrestrained liver glucose output and impaired peripheral glucose disposal. The ‘brake’ that insulin exerts on the breakdown of triglycerides in adipose tissue is also released, increasing fatty acid influx to the liver, fatty acid oxidation and synthesis of ketones, a reserve fuel for the brain normally only required in response to prolonged fasting. When lipolysis is completely unrestrained, liver supply of fatty acids grossly exceeds the demand of ketogenesis, and severe fatty liver and overproduction of triglyceride-rich lipoproteins ensues. Energy reserves in muscle are also raided, with progressive protein catabolism releasing amino acids for liver metabolism. The net result of these processes is ‘starvation in the midst of plenty’, the hallmark of untreated insulin deficiency.

In fully compensated IR, no clinical or biochemical dysregulation beyond increased size and activity of the pancreatic islets that synthesise insulin would be expected. However, this is not what is observed. The ‘common’ IR seen routinely in clinic, usually with obesity, is closely associated with metabolic dysregulation including fatty liver and increased blood lipid concentrations (dyslipidaemia) amongst a constellation of biochemical abnormalities.

Strikingly, many other abnormalities seen in compensated IR feature increased tissue growth (Reaven, 2005): Acanthosis nigricans (AN) is common in IR (Hermanns-Le et al., 2004), and denotes a brown, velvety skin appearance in flexures such as around the neck and under the arms. Microscopically, AN features benign hyperproliferations of cells at several levels of the skin (Torley et al., 2002). AN severity correlates with the severity of IR, and AN fades when beta-cell decompensation occurs, leading to a decrease in insulin concentration and, ultimately, diabetes. This suggests that AN is driven by high concentrations of insulin. Although sometimes a distressing manifestation of IR, AN is not a major cause of morbidity. However, the skin is not the only tissue in which hyperplasia and overgrowth are seen in response to IR.

In severe IR, pseudoacromegaly (Flier et al., 1993) is frequently seen, although the precise prevalence is not documented. This denotes a pattern of soft-tissue overgrowth that mimics acromegaly, a condition caused by excess growth hormone secretion in adults (Flier et al., 1993; Dib et al., 1998). Far more common is polycystic ovary syndrome (PCOS), which affects up to 10% of women and is associated with IR in 50-70% of cases (Goodarzi et al., 2011). PCOS is a major cause of subfertility (Goodarzi et al., 2011) and features increased ovarian volume and histological abnormalities, including hyperplasia of thecal cells and dyscoordinated follicular maturation (Jonard et al., 2007). Observations of people with single-gene causes of IR, or reversible severe IR due to INSR-blocking autoantibodies, establish that primary IR is sufficient to induce changes indistinguishable from PCOS, as long as secretion of gonadotrophin hormones from the pituitary gland is intact (Huang-Doran et al., 2021).

In the longer term, IR has been associated with increased cancer risk, and hyperinsulinaemia is one of the factors suggested to mediate the link between obesity and cancer (Pollack, 2007; Gallagher and LeRoith, 2020). In the case of oestrogen-dependent cancers, this may be explained through ovarian actions of insulin, which increase the duration of unopposed oestrogen action on tissues such as breast and endometrium (Nead et al., 2015). However, direct effects of high levels of insulin to promote cell growth and division are also likely to play a role. In the most severe forms of childhood IR caused by loss of INSR function, severe hyperplasia and overgrowth of the colon, ovaries and other organs, and hypertrophic cardiomyopathy is seen (Semple et al., 2011). Recently, there has also been increased focus on the notion that increased blood insulin concentrations may blunt the anticancer efficacy of inhibitors of growth factor signalling that also raise blood glucose (Hopkins et al., 2020).

What explains the apparent disconnect between compensation for IR with respect to blood glucose, and such a wide range of metabolic and tissue growth abnormalities? It is likely due to false assumptions commonly made about the negative feedback control of insulin action. Correction to ‘normality’ would require all of insulin's actions on different tissues to feature similar dose responses, and that they be subject to the same degree of negative feedback control as each other. Were this to be untrue, then correcting blood glucose with higher blood insulin concentration would exert potentially harmful effects on any ‘bystander’ tissues that remain relatively insulin responsive. This notion that compensatory hyperinsulinaemia may have adverse consequences on cardiometabolic outcomes, and that normalisation of plasma glucose may be achieved only at a significant cost, was first articulated by Gerald Reaven more than 30 years ago (Reaven, 1991).

“Correcting blood glucose with higher blood insulin concentration would exert potentially harmful effects on any ‘bystander’ tissues that remain relatively insulin responsive.”

## Chickens, eggs and human models

The initiating defect in common IR is unclear, and discriminating the cause and effect in the interconnected web of IR associations is extremely challenging. Often, causation is inferred from the temporal sequence in which abnormalities appear. However, human genetic variation offers one approach to test causation more formally, if genetic variants can be identified that cause only one element of a syndrome directly. Informative genetic variants may be single variants with large effects on one gene, such as rare loss-of-function mutations in *INSR*, or may be groups of more common variants with much smaller effect sizes individually, as identified in population-wide studies of IR (Scott et al., 2014; Yaghootkar et al., 2014). For current purposes, severe genetic defects that solely affect proximal insulin signalling, such as *INSR* defects, or selectively affect adipose tissue development or maintenance, a state known as lipodystrophy, are of particular value (Semple et al., 2011).

If a component of the ‘IR syndrome’ is caused solely by deficient insulin action, then it should be seen in undertreated type 1 diabetes. If it is caused by the compensatory hyperinsulinaemia that accompanies IR, then it will be exaggerated in people with severe IR due to *INSR* defects, as long as it depends on insulin signalling via a non INSR-dependent pathway. Conversely, if the IR syndrome component is reliant on INSR signalling, it will be attenuated. If a component of the IR syndrome is caused instead by adipose tissue dysfunction per se, it should be seen in lipodystrophy whether or not IR is present. Finally, if it requires IR as well as adipose dysfunction, then it will be seen in lipodystrophy with secondary IR but not in primary IR that is caused by signalling defects.

**Fig. 1.**
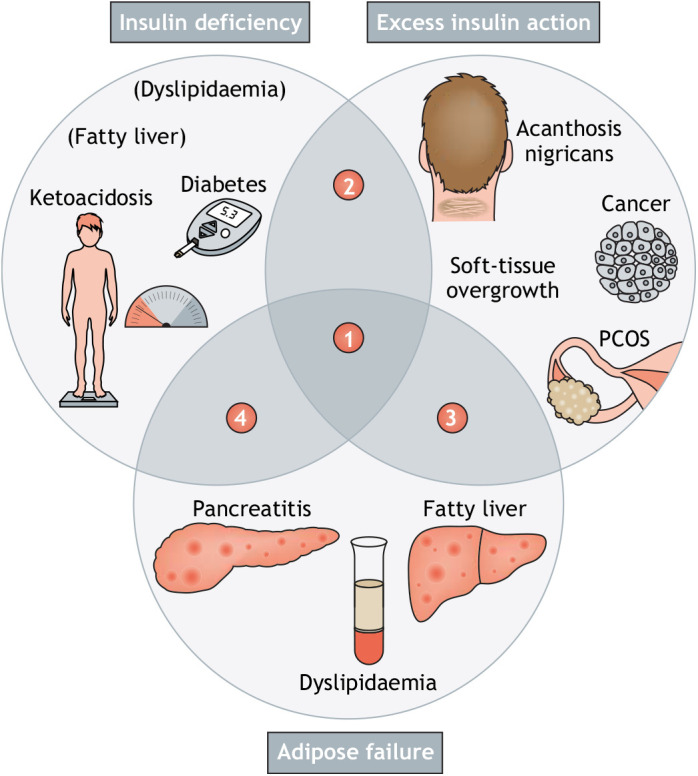
**The common insulin resistance syndrome and type 2 diabetes exhibit features of gain and loss of insulin action and adipose failure.** Features of insulin deficiency, excess insulin action and adipose failure are indicated, drawing on observations of untreated type 1 diabetes, fully compensated insulin resistance (IR) and lipodystrophy, respectively. Bracketed features are rare and only seen in the most severe degrees of insulin deficiency. Overlapping syndromes are numbered. (1) Characteristic of type 2 diabetes or lipodystrophy while insulin concentrations are still high. Some features from all three groups can be seen. (2) Seen in the early stages of decompensation of a proximal insulin signalling defect, e.g. due to INSR mutation. Features of excess insulin action with hyperglycaemia are most common; other features of insulin deficiency rarely develop due to residual insulin production. (3) Seen in obesity- or lipodystrophy-related IR before beta-cell decompensation. Commonly includes all features of lipotoxicity and excess insulin action. (4) Seen in more advanced stages of beta-cell decompensation in obesity- or lipodystrophy-related IR. Reversible features of excess insulin action disappear progressively as beta-cell failure progresses. PCOS, polycystic ovary syndrome.

Viewing obesity-related diabetes with IR and its associated abnormalities through this prism reveals three distinct drivers of abnormalities associated with common IR (Fig. 1). One group of abnormalities, all seen in untreated type 1 diabetes, such as hyperglycaemia and occasionally ketoacidosis, can be attributed to loss of insulin action. Another group features increased tissue and cellular growth in the form of AN, PCOS, pseudoacromegaly, cardiac hypertrophy and some cancers, and is attributable to primary IR with compensatory hyperinsulinaemia. In this group, many, and possibly all, of these problems disappear when beta cells fail, further implicating hyperinsulinaemia in their causation. The final group, which may broadly be categorised as ‘lipotoxic’, requires failure of adipose tissue homeostatic functions as well as IR (Fig. 1). Of note, fatty liver and dyslipidaemia are most extreme in this lipotoxic subset of IR features, but may also be caused by the most severe degrees of insulin deficiency. In this case, they are due to unrestrained adipose tissue lipolysis and massively increased delivery of free fatty acid to the liver. The protection from fatty liver seen in humans with severe IR due to *INSR* defects suggest that this, too, may be regarded as a consequence of excess insulin action in some circumstances (Semple et al., 2009), a contention supported by extensive evidence in mice (Brown and Goldstein, 2008).

It can thus be concluded that the wide range of clinical abnormalities seen in people with obesity-related type 2 diabetes with IR cannot all be attributed to loss of insulin action. Instead, they are a mixture of consequences of inadequate insulin action, excessive insulin action and adipose failure.

“Clinical abnormalities seen in people with obesity-related type 2 diabetes […] are a mixture of consequences of inadequate insulin action, excessive insulin action and adipose failure.”

## How does a compensatory increase in insulin cause disease?

The question of how increased blood insulin concentration causes tissue pathology in the context of IR then arises, as concomitantly increased and attenuated insulin action seems paradoxical. The concept of cell-autonomous ‘selective’ IR has been put forward as the simplest possible explanation for this, first in the context of the putative role of IR in promoting hypertension, endothelial dysfunction and vascular complications of diabetes. This was based on documentation of differential degrees of IR in different arms of the insulin signalling network in endothelial cells. Specifically, blood pressure-lowering, vasodilatory PI3K signalling was shown to be attenuated in IR, while blood pressure-increasing, vasoconstrictor RAS/ERK/MAPK signalling was enhanced (Potenza et al., 2005; Zeng and Quon, 1996; Hancock et al., 2019). This cell-autonomous explanation has also been widely discussed in the context of IR-associated metabolic abnormalities, and in particular the regulation of liver glucose and lipid metabolism by insulin in IR. According to this cell-autonomous interpretation of selective IR (Brown and Goldstein, 2008), insulin concentrations are set largely by its blood glucose-lowering ability, given the tight feedback that glucose exerts on pancreatic insulin secretion. Attenuation of insulin's glucose-lowering action first increases blood glucose and then insulin secretion until the glucose concentration is restored to the baseline level, at the expense of a higher blood insulin concentration. If any branch of the complex insulin signalling pathway is relatively unimpaired compared to the branch controlling glucose metabolism, however, the higher insulin concentration causes excessive activation of this pathway (Brown and Goldstein, 2008). This simple view can be refined by noting that it is not necessary to invoke a signalling defect that is truly selective to one branch of a pathway. A partial defect proximal to a branch point within the pathway could also, in principle, cause differential downstream effects if those branches have different dose responses to upstream stimulation (Parker et al., 2011; Cook et al., 2015).

Some evidence for truly pathway-selective IR, and also for differential downstream responses to a shared proximal signalling defect, has been advanced in mice and humans; however, metabolic focus has increasingly moved to a related but distinct interpretation of selective IR. This is viewed at the tissue level, rather than focusing on different insulin subpathways. In other words, insulin action on different tissues exhibits differences in ‘dose response’ with respect to the physiologically important effect, such as suppression of adipose triglyceride lipolysis, stimulation of muscle glucose uptake and suppression of liver gluconeogenic gene expression. Thus, even a fixed change in insulin signalling across all tissues may have different effects on tissue metabolism, and, as tissues are in constant metabolic dialogue mediated by substrate flux, this often will have systemic consequences. Because insulin levels are set predominantly by blood glucose concentration, the tissue that plays the biggest role in this will determine the insulin concentration that is sensed by other tissues. This sets the scene for potential exposure of bystander tissues to increased insulin action. It must also be remembered that many metabolic responses to insulin depend on substrate supply as well as signalling pathway activation. For example, although insulin activates liver gluconeogenic gene expression, a greater determinant of liver glucose output is delivery of substrates from adipose tissue, which is also under control of insulin (Titchenell et al., 2016; Vatner et al., 2015). Thus, the physiologically relevant action of insulin on liver glucose production is actually exerted predominantly on adipose tissue.

The role of selective IR in growth-related manifestations of IR has been addressed less directly, in large part due to the relative lack of these manifestations in mice. Some *ex vivo* studies of primary cells from patients suggested that a selective defect in ‘metabolic signalling’ via PI3K might explain excessive ovarian growth in the context of PCOS or pseudoacromegaly (Flier et al., 1993; Dib et al., 1998; Book and Dunaif, 1999; Corbould et al., 2006). However, people with primary defects impairing *INSR* function often have very-severe PCOS, arguing against this (Huang-Doran et al., 2021; Huang-Doran et al., 2016). Indeed, the notion that insulin exerts ‘metabolic’ effects via PI3K and ‘mitogenic’ effects mostly via RAS/RAF/MEK/ERK sits uneasily with the overgrowth caused by selective genetic activation of PI3K, and with the demonstrated crosstalk between the pathways (Madsen et al., 2018; Castellano and Downward, 2011). Although the assignment of binary roles to distinct pathways may be naïve, the concept is not wholly invalidated, and could be rehabilitated to take into account that different patterns of signalling may yield different biological outputs from the same pathway. In most cell signalling studies, the acute response of a signal-starved pathway to ligand exposure is assessed, but this fails to address kinetics of receptor downregulation and recycling, and signal extinction, which may in principle differ among tissues. These may become important determinants of insulin sensitivity in the face of high insulin concentrations *in vivo*.

## A tale of non-identical twins: IGF and insulin

The INSR is expressed as two isoforms, generated by alternate splicing of exon 11 (Seino and Bell, 1989). The B isoform, which includes exon 11, is thought to elicit primarily metabolic outcomes, while the A isoform promotes growth due to its high affinity for the insulin homologue insulin-like growth factor (IGF)-II (Kosaki et al., 1995). In common IR, the ratio of receptor isoform A to B is reported to be increased in metabolic tissues (Besic et al., 2015), and this has been suggested to underlie growth-related complications of IR such as cancer, as has been comprehensively reviewed elsewhere (Belfiore et al., 2017). Nevertheless, proving this is experimentally challenging, and much supporting evidence is correlational. Moreover, all naturally occurring *INSR* mutations described to date are common to both receptor isoforms, arguing against skewed INSR isoform function as the primary mechanism driving overgrowth and other consequences of genetic INSR dysfunction.

**Fig. 2.**
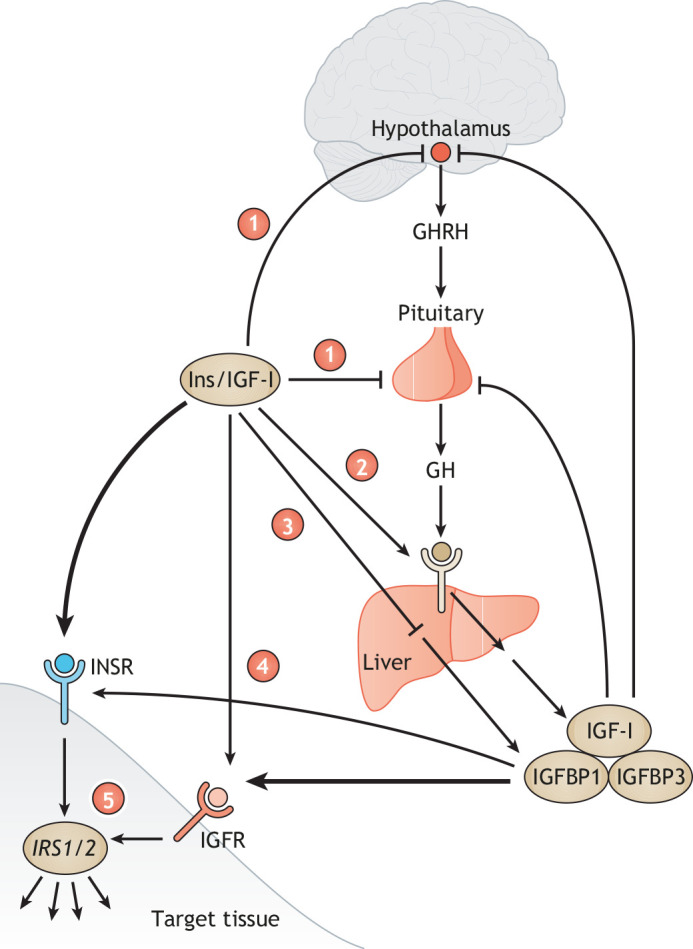
**Examples of endocrine and cellular crosstalk between insulin and growth hormone/IGF-I action.** (1) Insulin and IGFs exert negative feedback on growth hormone secretion at the hypothalamus, and pituitary (2) insulin upregulates hepatic growth hormone expression. (3) Insulin suppresses IGFBP1. (4) High concentrations of insulin or IGF-I can cross-activate each other's receptors. Hybrid insulin/IGF-I receptors will also be present and activated but are not shown here. (5) Close similarities of intracellular signalling cascades for insulin and IGF-I raise the possibility of mutual signalling potentiation. GH, growth hormone; GHRH, growth hormone-releasing hormone; IGF, insulin-like growth factor; IGFBP, IGF-binding protein; IGF-IR, insulin-like growth factor receptor; Ins, Insulin; INSR, insulin receptor; IRS, insulin-responsive gene.

In trying to disentangle metabolic and growth effects of insulin action, and how they are perturbed in disease, it may thus be necessary to look beyond insulin and its single canonical receptor (INSR), and to consider signalling by IGFs further (LeRoith et al., 2021). IGF-I is an endocrine hormone secreted by the liver, and a paracrine growth factor secreted by many tissues, positively regulated by growth hormone from the pituitary gland. IGF-I circulates in a complex with binding proteins that modulate its bioavailability. There is a high degree of crosstalk between insulin and the growth hormone–IGF-I action at multiple levels of physiological regulation (Fig. 2). Moreover, IGF-I has a cognate receptor homologous to the INSR, the insulin-like growth factor receptor (IGF-IR), and this can be stimulated by pathologically high insulin concentrations. Furthermore, the INSR and IGF-IR can heterodimerise to form so-called hybrid receptors, the existence of which is well established, but which have distinct (patho)physiological roles that are not fully elucidated (Siddle et al., 1994; Siddle, 2012). IGF-II, mentioned above, also circulates at high concentrations throughout life in humans, although much of it is sequestered in a ternary complex (Holly et al., 2019).

“In trying to disentangle metabolic and growth effects of insulin action, and how they are perturbed in disease, it may thus be necessary to look beyond insulin and its single canonical receptor (INSR).”

**Figure:**
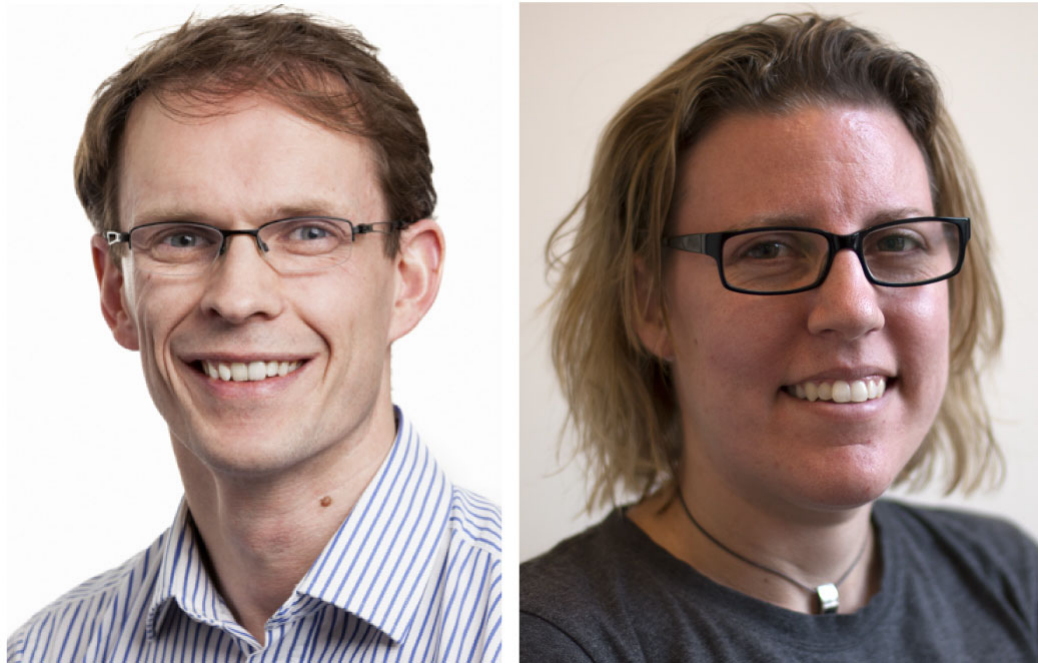
Robert Semple (left) and Gemma Brierley (right)

Decades of research have defined distinct actions of insulin and IGF-I *in vivo*, but attempts over many years to relate this to differences in signalling architecture have instead established that the signalling networks activated by the receptors are closely similar. Application of modern tools has suggested there are some quantitative differences in activation of particular elements of the network (Cai et al., 2017; Nagao et al., 2021; Liu et al., 2012), although many studies have used unphysiologically high ligand concentrations, sometimes orders of magnitude above interstitial concentrations. Collective findings, to date, suggest that ligand availability, tissue receptor expression profiles, expression of intracellular signalling components and, perhaps, distinct signalling dynamics may underpin the different biological roles of the receptors.

Given the limitations of reported mouse models discussed below, human genetic observations currently provide the best *in vivo* evidence for the interaction of insulin and IGF signalling in IR-related pathology. For example, infants with complete or near-complete loss of INSR function show striking soft-tissue overgrowth, including skin, ovaries, heart, colon, kidneys and other organs (Semple et al., 2011; Jospe et al., 1996), and this cannot be attributed to high levels of insulin signalling through its own receptor. In some cases of genetic INSR defects, derangement of IGF signalling has been directly demonstrated (Hojlund et al., 2012; Fowlkes et al., 2007). Conversely, various primary disorders of IGF production or action are reported to perturb insulin sensitivity (e.g. Gannage-Yared et al., 2013; Mohn et al., 2011; Murphy et al., 2012; Douillard et al., 2020; Domené et al., 2011), while IGF-I is frequently used as therapy for patients with extreme recessive IR due to INSR mutations (McDonald et al., 2007).

Abundant human genetic and biochemical evidence thus argues that dysregulation of the complex interplay between insulin and IGF signalling plays an important role in pathogenesis of several long-term complications of IR, particularly relating to excess tissue growth. Further long-term study of the reciprocal growth and metabolic complications in human monogenic disorders may yield further insight into the potential risks and benefits of strategies aimed at dual modulation of insulin/IGF-I signalling in IR.

## Shortcomings of current models

Mouse models of acquired and genetic IR are valuable tools for mechanistic dissection of human disease. However, there are many important quantitative differences between mice and humans in trajectories and regulation of development and growth, and in configuration and hormonal regulation of metabolism. Where like-for-like comparisons are possible between human monogenic disorders and mouse models, similarities do not always appear. Unlike humans with complete loss of *INSR* function, who live with growth impairment and IR but not ketoacidosis for several months, *Insr* knockout mice die of ketoacidosis within hours of birth (Joshi et al., 1996; Accili et al., 1996; Nakae et al., 2001). Human generalised lipodystrophies are fairly well modelled by knockout mice (Rochford, 2014), but for human inherited partial lipodystrophy, correlation between human and mouse phenotypes is rather poor, a problem compounded by a lack of germline knock-in models for some of the most common human lipodystrophies.

There are also important human–murine differences pertinent to the interplay of insulin and IGFs in disease and their end-organ effects. For example, mice do not have high circulating concentrations of IGF-II postnatally, whereas even adult humans have very-large circulating reservoirs (LeRoith et al., 2021; Holly et al., 2019). The insulin-responsive gene sex hormone-binding globulin (*SHBG*) links hyperinsulinaemia and altered testosterone concentrations in humans, with genetic studies suggesting a bidirectional relationship between SHBG and metabolic disease (Perry et al., 2010; Semple et al., 2008), yet no orthologue circulates in mouse plasma (Holly and Perks, 2012). Even in mouse models that do exhibit severe IR, no robust phenotype corresponding to human PCOS has been described (Huang-Doran and Franks, 2016). With the exception of a single report implicating ectopic activation of cardiac IGF signalling in a murine genetic model of generalised lipodystrophy (Zhou et al., 2019), other aspects of tissue overgrowth have not been studied in mouse models, to our knowledge.

Cellular studies bypass much of the complexity inherent in an interconnected physiological system. Nevertheless, technical limitations constrain signalling studies. Many immortalised cell lines have levels of IGF-IR, IGF and IGF-binding protein (IGFBP) expression that differ from those of native cells, which complicates studies of insulin and/or IGF action. Although the existence of INSR/IGF-IR hybrids has been documented in many cells and tissue types, much of what is believed about their physiological importance is inferred from circumstantial observations in the absence of tools that selectively perturb hybrid formation but not receptor homodimerisation (Siddle, 2012).

In summary, the complexity of the dual insulin/IGF-I signalling system, the multifaceted differences in its constitution between humans and rodents, and apparent differences in the propensity of the different species to exhibit consequences of IR, complicate efforts to model and dissect long-term IR complications in mice. Further genetic and environmental humanisation of murine models may be possible, but, at present, studies of human ‘genetic models’ may offer the more reliable window into common disease mechanisms.

## Future directions

Medical approaches to obesity-related IR to date have tended to focus on diabetes, dyslipidaemia and, to a lesser extent, on other metabolic complications such as fatty liver. Nevertheless, for many people, consequences of IR including ovarian dysfunction and subfertility, or soft-tissue overgrowth, either benign or malignant, are major concerns, and current evidence implicates dysregulated IGF action in their pathogenesis in the context of IR. We thus suggest that a more nuanced view of IR may help the metabolic field to address the multifaceted challenges posed by the modern obesity pandemic. It may be time to relinquish the simple endocrine paradigm of one hormone and one receptor and a binary tissue response. It could be of value to consider insulin and IGF production and action as inter-related, rather than as discrete systems that exhibit crosstalk. It will also be increasingly necessary to address the way in which distinct outcomes may be encoded by different spatiotemporal patterns of signalling even by the same receptor, and how this is perturbed in disease by alteration of the dual insulin/IGF system. Finally, the concept of ‘selective IR’ should be solidified and interpreted at an organismal as well as cellular level. It may then be possible to develop therapeutic strategies that rationally rebalance insulin signalling through selective modulation of both insulin and IGF signalling, rather than simply increasing global insulin action.
